# ACCESS TO EVERYDAY ACTIVITIES IN PUBLIC SPACE - VIEWS OF PEOPLE WITH DEMENTIA

**DOI:** 10.1093/geroni/igz038.2847

**Published:** 2019-11-08

**Authors:** Anna Brorsson, Louise Nygard, annika Ohman

**Affiliations:** 1 Division of Occupational Therapy, NVS, Karolinska Institutet, Huddinge, Stockholms Lan, Sweden; 2 Division of Occupational Therapy, Department of Social Welfare Studies. Linkoping University, Norrkoping, Ostergotlands Lan, Sweden

## Abstract

People with dementia value staying active and continuing with their everyday lives in public space, however there is a lack of knowledge about how they experience accessibility, problematic situations and how to manage these situations. The aim is to illuminate experiences of accessibility in public space in people with dementia with focus on places, activities and problematic situations. A Grounded theory approach was used in the thesis with multiple data collection methods (interviews, focus group interviews, observations and visual methods). Findings show that having access to everyday activities at different places in the neighbourhood was very important for the participants when they perceived themselves as being a part of the society and being active and independent persons. Engaging in familiar activities in familiar places was important. However, their activity radii in the community became smaller. The findings inspired the development of the questionnaire Participation in Activities and Places Outside Home.

